# Supplementary figures and images for: Human tonsil organoids reveal innate pathways modulating humoral and cellular responses to ChAdOx1
Source: PLoS Pathog. 2025 Aug 22;21(8):e1013432. doi: 10.1371/journal.ppat.1013432 (PMC12393735; doi:10.1371/journal.ppat.1013432)

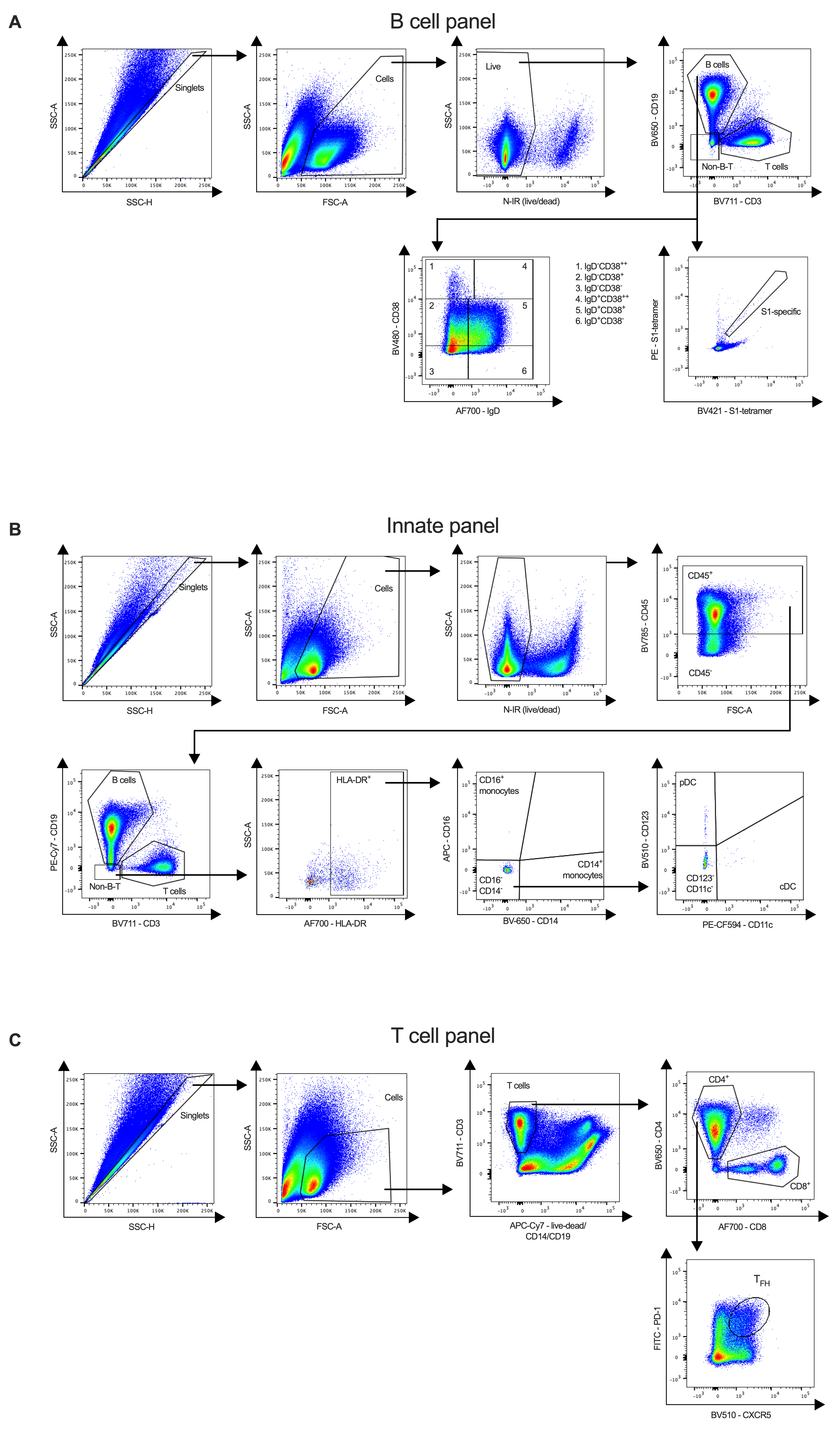

Supplement: S1 Fig — Gating strategy for (A) B cell, (B) innate, and (C) T cell panels. (TIFF) [file ppat.1013432.s002.tiff]

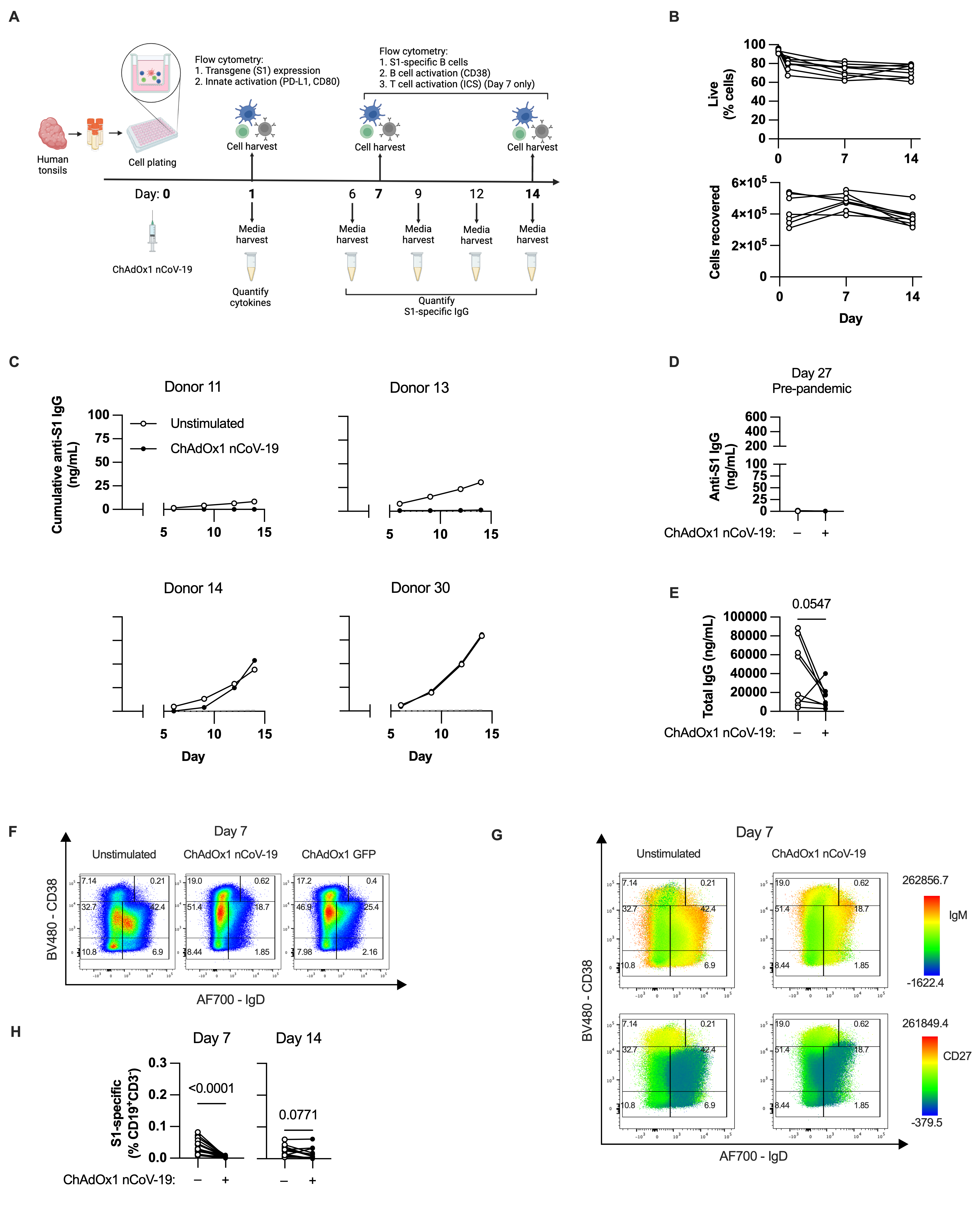

Supplement: S2 Fig — (A) Experimental schematic of tonsil organoid culture system. Schematic was generated in BioRender (https://BioRender.com/3a5hazz). (B) Viability and cell recovery from tonsil organoid cultures over time. (C) Cumulative anti-S1 IgG production over time in donors with spontaneous anti-S1 IgG production. (D) Anti-S1 IgG production in tonsil organoids from pre-pandemic donors after 27 days of culture with or without ChAdOx1 nCoV-19 stimulation. (E) Cumulative total IgG production in post-pandemic tonsil organoids with or without ChAdOx1 nCoV-19 stimulation after 14-day culture. (F) Representative FACS plots of IgM and CD27 expression in B cell subsets from unstimulated and ChAdOx1 nCoV-19-stimulated organoids on day 7 of culture. (G) Representative FACS plot of B cells from unstimulated, ChAdOx1 nCoV-19-stimulated, and ChAdOx1 GFP-stimulated organoids on day 7 of culture. (H) Percentage of S1-specific B cells in unstimulated and ChAdOx1 nCoV-19-stimulated organoids from post-pandemic donors. Data in B are combined from 2 experiments with 9 donors, D from 1 experiment with 4 donors, E from 2 experiments with 8 donors, H from 4 experiments with 16 donors. Each symbol represents an individual donor. Values in D, E, and H were compared using Wilcoxon matched pairs signed rank test. (TIFF) [file ppat.1013432.s003.tiff]

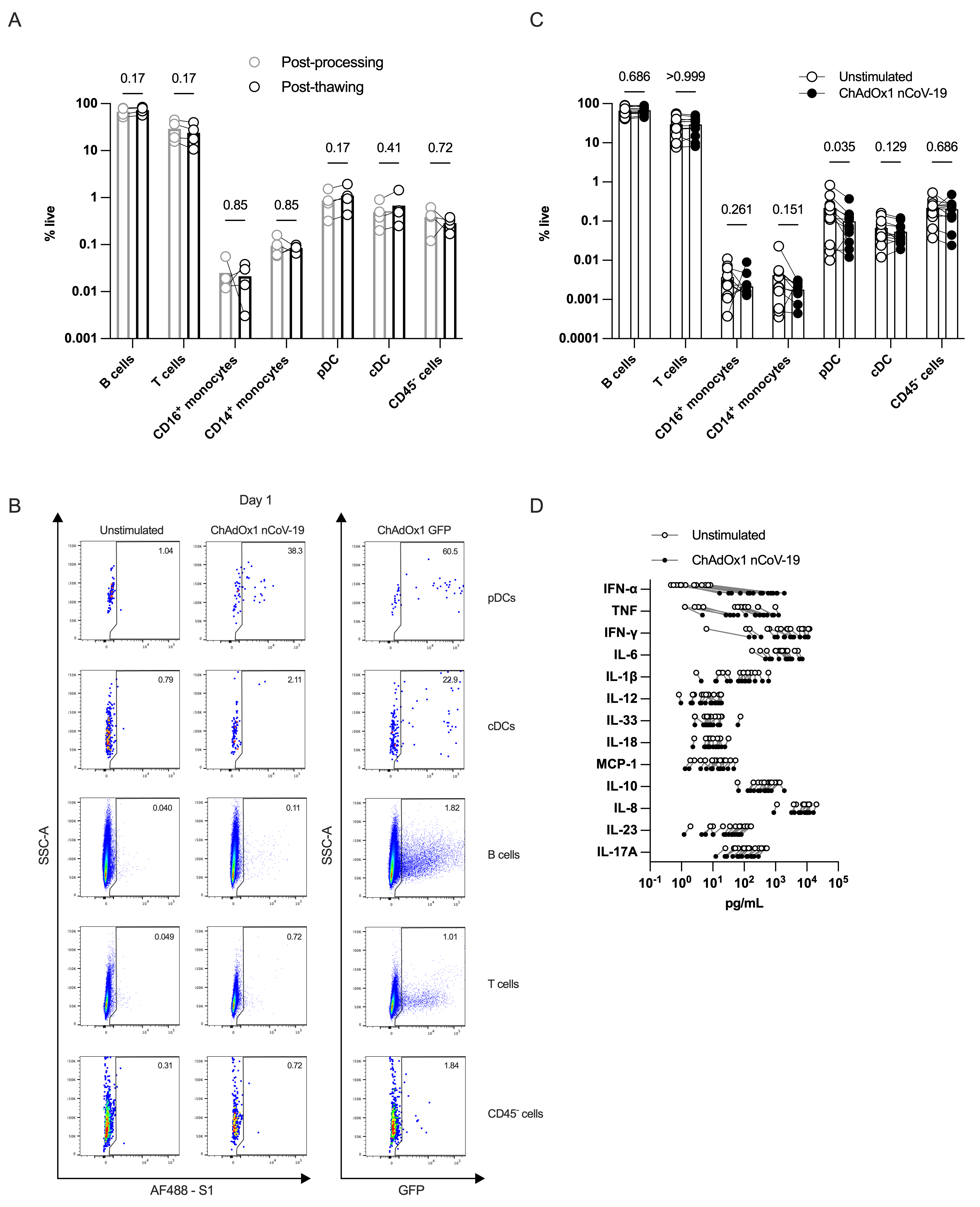

Supplement: S3 Fig — (A) Composition of cell types recovered after tonsil processing compared to after thawing frozen tonsil cells. (B) Representative FACS plots of ChAdOx1 transgene expression (either S1 spike or GFP) for each cell type. (C) Composition of cell types recovered from unstimulated and ChAdOx1 nCoV-19-stimulated tonsil organoids after 24 hours in culture. (D) Cytokine levels in media supernatants from unstimulated and ChAdOx1 nCoV-19-stimulated tonsil organoids after 24 hours in culture. Data in A are from 1 experiment with 4 donors, C from 3 experiments with a total of 12 donors, D from 4 experiments with a total of 16 donors. Each symbol represents an individual donor. Values in A were compared using multiple t-tests, values in C were compared using multiple Wilcoxon tests. (TIFF) [file ppat.1013432.s004.tiff]

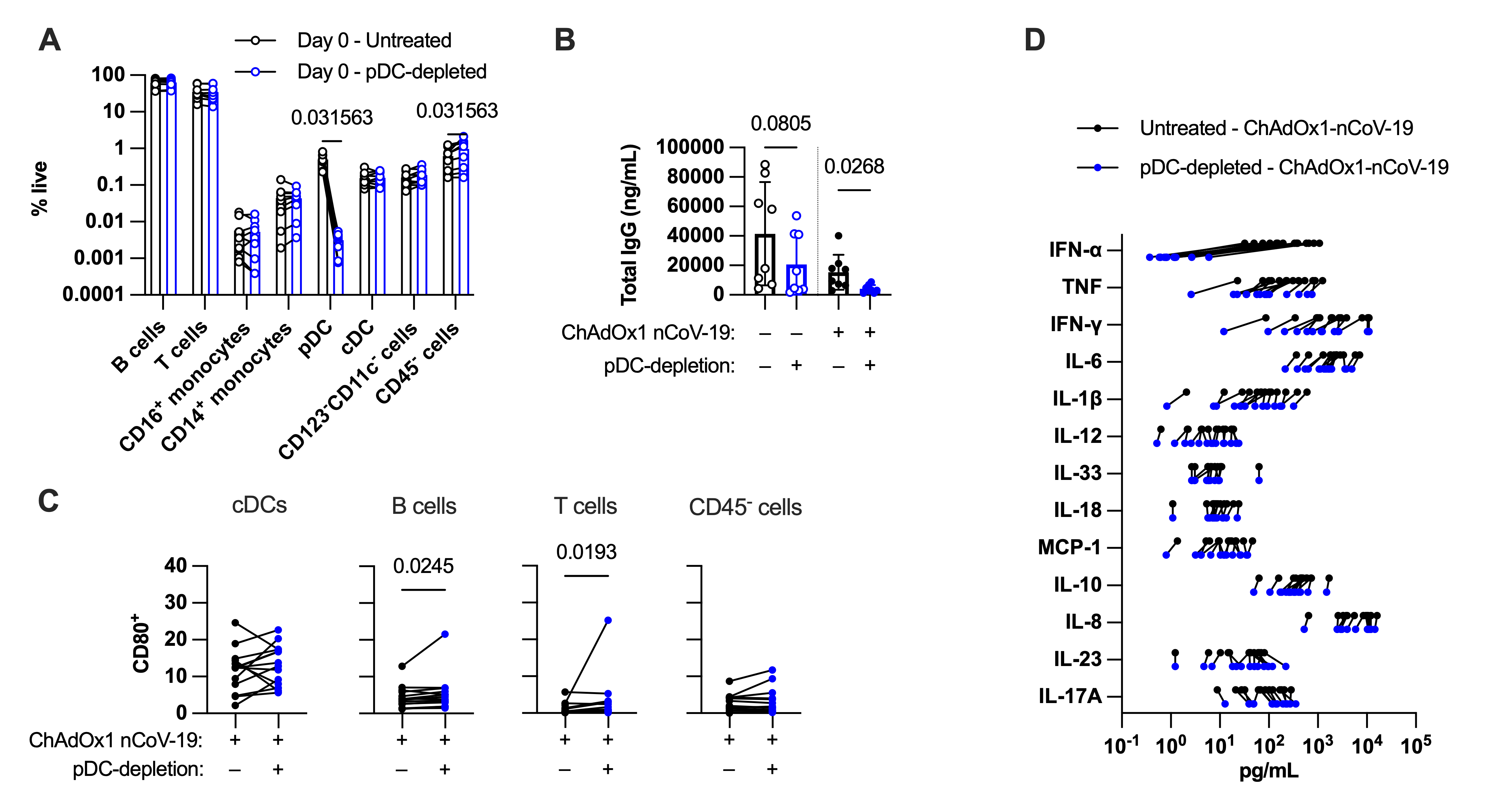

Supplement: S4 Fig — (A) Composition of cell types recovered from undepleted and pDC-depleted tonsil cells before plating. (B) Cumulative total IgG production in unstimulated or ChAdOx1 nCoV-19-stimulated organoids with or without pDC-depletion after 14-day culture. (C) Surface CD80 expression on different cell types in ChAdOx1 nCoV-19-stimulated tonsil organoids with or without pDC-depletion after 24 hours in culture. (D) Cytokine levels in media supernatants from ChAdOx1 nCoV-19-stimulated tonsil organoids with or without pDC-depletion after 24 hours in culture. Data in A are from 3 experiments with a total of 9 donors, B from 2 experiments with a total of 8 donors, C-D are combined from 4 experiments with a total of 14 donors. Each symbol represents an individual donor. Values in A were compared using multiple Wilcoxon tests, values in B were compared using Friedman test with Dunn’s multiple comparisons test, values in C were compared using Wilcoxon matched pairs signed rank test. (TIFF) [file ppat.1013432.s005.tiff]

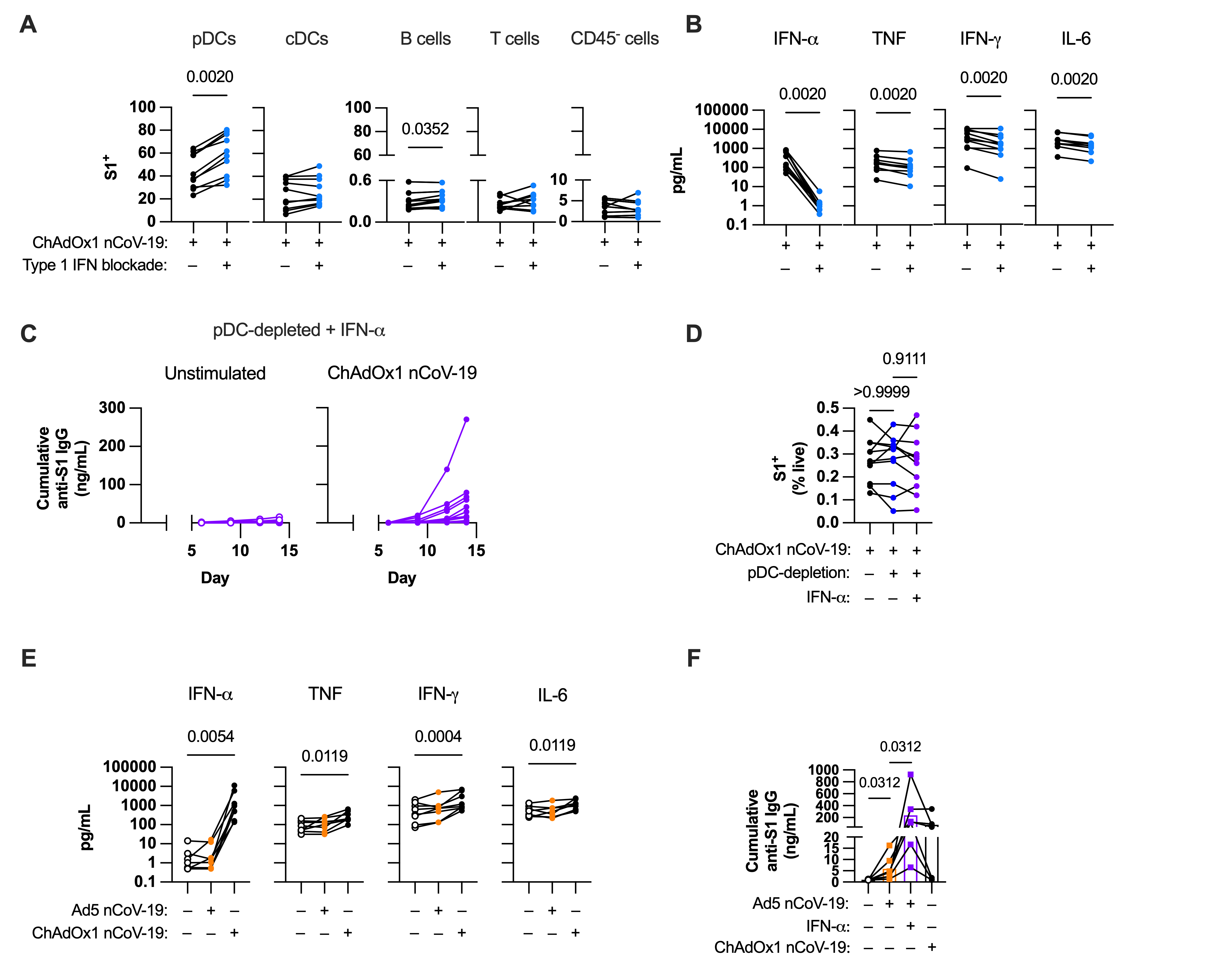

Supplement: S5 Fig — (A) Transduction rates of different cell types in ChAdOx1 nCoV-19-stimulated tonsil organoids with or without type 1 IFN blockade after 24 hours in culture. (B) Levels of IFN-ɑ, TNF, IFN-γ, and IL-6 in ChAdOx1 nCoV-19-stimulated tonsil organoids with or without type 1 IFN blockade after 24 hours in culture. (C) Cumulative anti-S1 IgG production in post-pandemic tonsil organoids with pDC-depletion and IFN-α supplementation over 14-day culture. (D) Percentage of total transduced cells after 24-hour culture in ChAdOx1 nCoV-19-stimulated tonsil organoids with or without pDC-depletion and IFN-α supplementation. (E) Cytokine levels in media supernatants from tonsil organoids stimulated with Ad5 nCoV-19 or ChAdOx1 nCoV-19 after 24 hours in culture. (F) Cumulative anti-S1 IgG production in tonsil organoids stimulated with Ad5 nCoV-19 with or without IFN-α supplementation or ChAdOx1 nCoV-19 after 14-day culture. Data in A-B are combined from 3 experiments with 10 donors, C and D from 3 experiments with 11 donors, E from 1 experiment with 8 donors, F from 1 experiment with 7 donors. Each symbol represents an individual donor. Values in A and B were compared using Wilcoxon matched pairs signed rank test, values in D-F were compared using Friedman test with Dunn’s multiple comparisons test. (TIFF) [file ppat.1013432.s006.tiff]

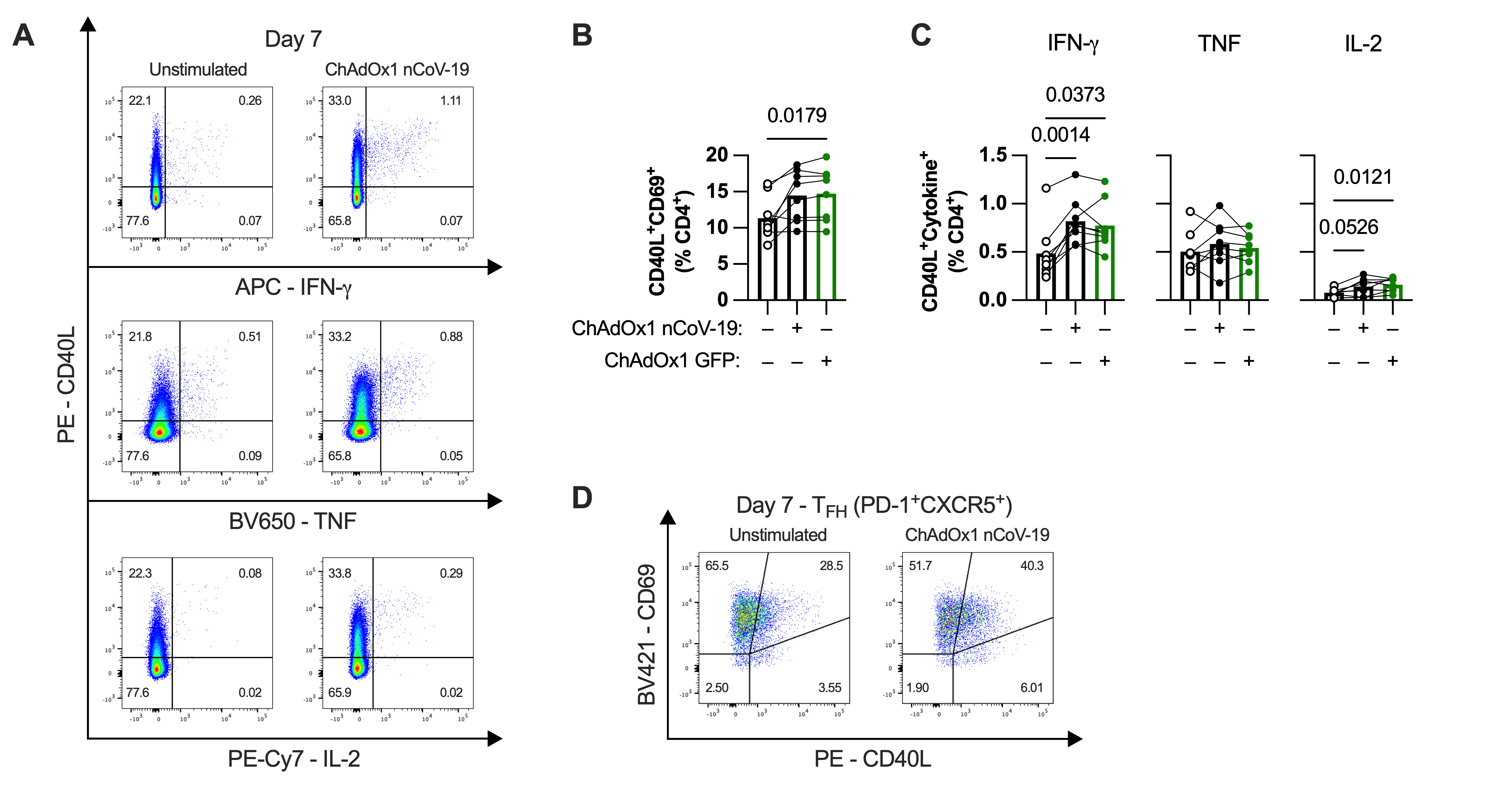

Supplement: S6 Fig — (A) Representative FACS plots of CD40L and intracellular cytokine (IFN-γ, TNF, and IL-2) expression in CD4+ T cells in ChAdOx1 nCoV-19-stimulated tonsil organoids. (B-C) Percentage of CD40L+CD69+ cells (B) and co-expressing CD40L and cytokines (C) of CD4+ T cells in tonsil organoids stimulated with ChAdOx1 nCoV-19 or ChAdOx1 GFP. (D) Representative FACS plots of CD69 and CD40L expression on TFH (CD4+PD-1+CXCR5+) cells in ChAdOx1 nCoV-19-stimulated tonsil organoids. All results were from cell harvest at 7 days after plating. Data in B and C are from 1 experiment with a total of 8 donors. Each symbol represents an individual donor. Values in B and C compared using Friedman test with Dunn’s multiple comparisons test. (TIFF) [file ppat.1013432.s007.tiff]
