# Supplementary material for: Human tonsil organoids reveal innate pathways modulating humoral and cellular responses to ChAdOx1
Source: PLoS Pathog. 2025 Aug 22;21(8):e1013432. doi: 10.1371/journal.ppat.1013432 (PMC12393735; doi:10.1371/journal.ppat.1013432)
Supplement: Attachment — Submitted filename: Rebuttal.docx [file ppat.1013432.s009.docx]

**Reviewers' Comments:**

Reviewer's Responses to Questions

**Part I - Summary**

Please use this section to discuss strengths/weaknesses of study, novelty/significance, general execution and scholarship.

Reviewer #1: This manuscript describes the innate immune response to a chimpanzee adenovirus-vectored vaccine for SARS-CoV-2 (ChAdOx1 nCoV-19) using a human tonsil organoid model. The main conclusions of the paper are that AdV immunogenicity is associated with the innate activation of PDCs, which are the primary producers of T1 IFNs in the model. The authors also show that T1 IFN supports AdV responses by inducing IL-6 production, which can promote T follicular helper responses. For the most part, the experiments are rigorously designed and data presented are transparent. In my opinion, the findings represent an incremental advance to our existing understanding of the pDC/B cell axis and the roles of T1 IFN and IL-6 in these responses. T1 IFN responses to viral vectors are well described (https://doi.org/10.1016/j.ymthe.2020.01.001; https://doi.org/10.4049/jimmunol.179.3.1721; https://doi.org/10.1172/JCI37607) and the role of pDCs in T1 IFN production in tonsil organoids is shown in the paper describing the model (https://doi.org/10.1038/s41591-020-01145-0). The downstream associated connections, that T1 IFNs support AdV responses through IL-6, which can promote CD4 T cell responses, has also been described previously (https://doi.org/10.1006/mthe.2002.0658 as an example). Given that the main take-homes have been described in other publications, I’m not convinced that sufficient novelty has been demonstrated.

Reviewer #2: In this article, Pudjohartono et al. work towards investigating the immune responses directed against ChAdOx1 using human tonsil organoids. The authors show that upon exposure of post- SARS-CoV2 pandemic tonsillar organoids, spike specific antibodies increase over time within tonsillar organoids, with an increase in spike specific antigen secreting B-cells. In addition to increase in several immune cell subsets, notably pDCs, there was also an increase in several cytokines such as IFN-a, TNF and IL-6, with decrease in cytokines such as IL-17a, IL-23 when compared to unstimulated controls. The increase in pDCs was shown to be crucial for the secretion of spike specific antibodies, with the IFN-a and IL-6 shown to augment B and Tfh cell responses.  The article is well written, succinct and the authors have clearly shown the important role of pDCs in generation of spike specific specific antibodies when exposed to ChAdOx1, with adenovirus based vaccines showing similar immune responses when used for other infections. There are a few concerns however that need to be addressed, namely the exact mechanism of how cytokines regulate antibody product

Reviewer #3: Pudjohartono et al utilized a human tonsil organoid model to study the regulation of adaptive responses to ChAdOx1 nCoV-19. They documented early innate immune activation and cytokine release and subsequent late T and B cell activation and antigen-specific antibody secretion. They further show that plasmacytoid dendritic cells (pDCs) are transduced the most and their IFN- α secretion was critical for humoral responses and IL-6 enhanced it. The authors present an interesting model to gain mechanistic insights into vaccines in vitro. The work is interesting and provides a new avenue to model vaccine research thereby warranting publication. Few minor points are suggested to improve the manuscript.

**Part II – Major Issues: Key Experiments Required for Acceptance**

 Please use this section to detail the key new experiments or modifications of existing experiments that should be absolutely required to validate study conclusions.  Generally, there should be no more than 3 such required experiments or major modifications for a "Major Revision" recommendation. If more than 3 experiments are necessary to validate the study conclusions, then you are encouraged to recommend "Reject".

Reviewer #1: I provide a few suggestions for strategies to improve the novelty of the work.

1) Antibody non-responders were discarded from the study early on. It would be potentially interesting to determine if their innate responses (T1 IFN and IL-6) were different than the responders, and if so, how.

Response:

Thank you for the observation on this possible ambiguity. We included all donors that fulfilled the inclusion criteria of elective tonsillectomy, excluding individuals receiving immunosuppressive treatment, with severe inflammatory or exudative lesions on the tonsils, or with underlying immunocompromising disease.

We then excluded any donors with spontaneous anti-S1 IgG production at baseline from further analyses, as this spontaneous production was associated with suppression of responses upon stimulation and thus, vaccine responses could not be modelled (lines 241-242). However, donors with no detectable antibody production in response to stimulation (who also did not spontaneously produce antibody) were still included in further experiments. These non-responders were retained because we feel they reflect the natural variation in responses seen in humans following vaccination (i.e. vaccine non-response occurs in vivo). We have revised our wording (lines 243-244) for greater clarity.

We did not observe any difference in innate immune responses specifically associated with individuals who did not produce antibody in the assay system. As noted in the new Fig 1H, there is a strong correlation between frequency of pre-existing S1-specific memory B cells and the strength of antibody response upon stimulation. Therefore, we believe this variation in memory levels accounts for the differences in antibody responses, as opposed to major differences in innate immune responses.

Amendment:

- Revised text (lines 243-244) to clarify that donors with spontaneous anti-S1 IgG production at baseline were excluded from further analyses

2) One could imagine that different AdV might be more or less stimulatory of innate responses through T1 IFN and IL-6. Identifying vector-specific differences in the tuning for these cytokines could be of interest, as well as how those differences influence the down-stream B cell and T cell responses.

Response:

We agree that it would be interesting to compare to ChAdOx1 responses to a different AdV. We tested this using human adenovirus 5 (Ad5) encoding the SARS-CoV-2 spike protein (same antigen as ChAdOx1).

In accordance with previous literature on human PBMCs (Teigler *et al.*, J Virol, 2012; Provine et al., Science, 2021), Ad5 did not induce type 1 IFN nor IL-6 in the experimental model (Fig S5E). Ad5 induced minimal anti-S1 IgG compared to ChAdOx1 nCoV-19 (Fig S5F). However, adding type 1 IFN to the culture system augmented Ad5-induced specific antibody production (Fig S5F). We concluded that type 1 IFN is a major regulator of AdV-induced antibody production in the tonsil organoid model, as shown by the regulation of both Ad5 and ChAdOx1-induced responses.

Amendment:

- Added figures (Fig S5E-F) and text (lines 379-387) on Ad5 as a comparison adenovirus vector

Reviewer #2:

1. It would be informative for the readers to provide the gating strategy (or strategies) used to define cell populations in the study (can provide this as a supplementary figure).

Response:

Thank you for this suggestion, we have added the gating strategies used in Fig S1. We have also added revised the relevant lines in the Results section to refer to the gating strategies.

Amendment:

- Added Fig S1 and text (line 195) to show gating strategies used in the study
- Amended text (lines 266, 304, 428) to refer to gating strategies

2. The lines 233-234, the authors state that there was higher CD27 expression when compared to other subsets. Is this comparison within the subsets (i.e. the other gated populations) of the ChAdOx1 treated cells or when compared to the unstimulated cells? Based on Figure S1D (lower panel), it seems like CD27 expression is higher IgD-CD38++ populations in unstimulated cells when compared to ChAdOx1 treated cells. Please clarify.

Response:

Thank you for highlighting this ambiguous phrasing. We meant that the IgD^-^CD38^++^ subset had higher CD27 expression compared with the other B cell subsets within the same condition. This finding was consistent across both unstimulated and ChAdOx1-stimulated wells (Fig S1G).

The Reviewer is correct that average CD27 expression of the IgD^-^CD38^++^ subset is higher in the unstimulated cells compared to the ChAdOx1-treated cells. Recently activated memory B cells upregulate CD27, thereby converting from CD27^+^ to CD27^++^, during plasmablast differentiation (Avery *et al.*, J Immunol, 2005). Since the IgD^-^CD38^++^ population is expanding in the ChAdOx1-treated cells as memory B cells convert to plasmablasts, we therefore interpret these data as the entry of these recently activated cells (that have yet to convert to CD27^++^) into the gate, which results in a lower average CD27 expression as compared to the smaller pre-existing IgD^-^CD38^++^ cell population present in the unstimulated condition.

Amendment:

- Modified text to clarify comparison of CD27 expression within subsets in the same condition (lines 275-277)
- Added discussion on CD27 expression in IgD^-^CD38^++^ subset with ChAdOx1 stimulation (lines 277-281)

3. While the amount of transduction rates are quite low, can the authors speculate as to why transduction rates in T-cells are significantly lowered when pDCs are depleted in ChAdOx1 treated samples (Figure 3D, T-cells)?

This is an interesting question. We do not currently understand how T cells are transduced by Ad vectors, or why this occurs given the clear inefficiency of the process. The data from pDC depletion presents an interesting hypothesis. PD-L1 is upregulated on T cells in response to stimulation (Fig 2C), and this is regulated by pDCs/type I IFN (Fig 3C & 4E). As PD-L1 upregulation is a marker of cellular activation (not to be confused with cognate T cell activation), we speculate that this manner of activation may increase the transducibility of T cells by an undefined mechanism. If this model is correct, then the depletion of pDCs, which in turn reduces activation of the T cells, would result in reduced transduction, which we observe. As we currently lack any data directly testing this model, we worry it would be distracting to the reader to include a discussion on this point so have not included this speculation in the revised manuscript. We hope the Reviewer agrees with our decision.

 4. Based on Figure 5, it is still unclear what cell produces IL-6 in the organoid model. Given the importance of IL-6 in generating S1 specific antibodies (Figure 5B), can the authors speculate what is the source of IL-6 in organoids ? The source of IL-6 is still something that is important to figure out. What is the reason for IL-6 secretion to increase when B-cells are depleted from the tonsillar organoids.

Response:

Thank you for bringing this point to our attention. We have added a summary of IL-6 expression rates across the different cell types (Fig 5F) and the proportion of cell types accounting for the total IL-6^+^ cells (Fig 5G). Although all cells appear to produce IL-6 to some extent, the highest IL-6 expression rates are found in classical dendritic cells (Fig 5F). However, B cells dominate the overall IL-6-expressing cell counts (Fig 5G), which is due to the high frequency of B cells in the organoid culture system (Fig S3A). Other non-B cells (especially cDCs) may have higher rates of IL-6 production but are out-numbered by B cells in the undepleted culture system.

Since depleting B cells increases the proportional number of other cell types in the culture (as the input cell number is kept constant), B cell depletion appears to increase IL-6 secretion because there are proportionally more non-B cells present in the culture. We have added a paragraph in the discussion highlighting this important point and thereby caution against overinterpretation of the data.

Amendment:

- Added figures (Fig 5F-G) and revised text (lines 406-412) to summarise IL-6 expression in different cell types and contribution of each cell type to total IL-6^+^ cells
- Added text to the discussion (lines 545-553) discussing the limitations when interpreting these data due to technical aspects of the experimental design

5. The authors clearly show the importance of IFNa and IL-6 in regulating immune responses towards ChAdOx1. In addition to these two cytokines, TNF is another cytokine that is found abundantly in ChAdOx1 treated samples and significantly affected by pDC depletion. Have the authors tried performing depletion of TNF to see its effect of antibody production and cellular activation ?

We agree that TNF would be interesting to test, but we have not examined the effect of TNF blockade in the system at this time. Based on the proof-of-concept demonstrated in the current study, we intend to more systematically examine cytokines, including TNF, in the regulation of these vaccine responses. We hope the reviewer will agree the current manuscript is sufficiently comprehensive in its revised form to stand as a complete publication.

 6. Figure 8 is a little confusing as a pathway of the immune responses and cells involved is not clear. Does IL-6 directly affect B-cell activation or does it work via Tfh regulation ? What are the exact non B-cells that play a role in adenovirus based vaccine immunity ?

The feedback on the confusing nature of the model is appreciated. We have simplified the model (Fig 8) to remove the more speculative aspects of cell-cell signalling, which hopefully increases clarity. While it is intuitive that Tfh cells would be providing signals to the responding B cells, at this time we have no data to support (or refute) a role for T cell help in these B cell responses. Therefore, we have removed this dashed connecting arrow. Given a clear literature for IL-6 directly promoting plasmablast activation (Jego *et al.*, Blood, 2001) we believe that direct activation must be a mechanism of action. Additionally, based on new data in Fig 5, we have increased clarity that the “non-B cell” population responsible for IL-6 production is in-fact primarily cDCs. The model has been updated to reflect these data. Hopefully this combination of changes helps improve interpretability.

Amendment:

- Modified Fig 8 to reflect our revised and simplified model

Reviewer #3: None

**Part III – Minor Issues: Editorial and Data Presentation Modifications** Please use this section for editorial suggestions as well as relatively minor modifications of existing data that would enhance clarity.

Reviewer #1:

Fig 3: pDC depletion: it is not a novel finding that pDCs, through T1 IFN and IL-6, are required to support a humoral response (Jego et al., Immunity 2003). It is also not a novel finding in the model used by the authors (Wagar et al., Nature Medicine 2021).

We thank the Reviewer for the feedback to clarify the novelty of our findings. We appreciate that the emphasis of our claims of “novelty” were perhaps not sufficiently clear, as we readily acknowledge that both Type I IFN and IL-6 have well-reported roles in regulating humoral immunity. Instead, we feel the new findings presented in this context are how these cytokines specifically relate to Ad vectors and how these processes differ from what has been previously reported. We appreciate the Reviewer drawing to our attention the seminal findings of Jego *et al.,* which we now include in the discussion. As stated, Jego *et al.* have reported roles for Type I IFN and IL-6 in regulation of humoral immunity using an in vitro model. However, a major difference between this report and our current findings is how IL-6 production is modulated by pDCs. Jego *et al.* found in their reductionist system that IL-6 was produced directly by pDCs. However, in our system, we do not have evidence of IL-6 production by pDCs and instead find that pDCs modulate IL-6 production indirectly through cDCs as an intermediate producer cell type. We have explicitly added this comparison to the discussion.

The findings of Wagar *et al.* with relation to a role for pDCs is mentioned (lines 498-500; ref 13), and we state that our findings extend their original conclusions to a new, and biologically distinct, vaccine technology.

Amendment:

- We have added a major point to the discussion (lines 517-523) comparing our current findings with the prior findings of Jego *et al.*

Fig 3/4: The authors show that SARS-CoV-2 specific antibodies are decreased when pDCs are depleted or T1 IFN is blocked. What about total (non-specific) antibody production?

Response:

We agree that the question about non-specific antibody production is an interesting question to address. We measured the total IgG production in cultures with/without ChAdOx1 nCoV-19 stimulation and pDC-depletion. Interestingly, ChAdOx1 nCoV-19 stimulation decreased total IgG production (Fig S2E), possibly due to resource competition among recently activated B cells.

pDC-depletion decreased total IgG production in both unstimulated and ChAdOx1-stimulated cultures (Fig S4B), suggesting that the pDC type 1 IFN axis contributes to total IgG production in general.

Amendment:

- Added figures (Fig S2E, S4B) and text (lines 343-344) on effect of pDC-depletion on total IgG production

Fig 5: The interpretation of these data are complicated by the possibility that cells under various selection and depletion conditions might have different preferences for using up the secreted cytokines (i.e., that there could be influences on both the secretion and the usage side).

Response:

This is an important caveat to the interpretation of our conclusions, which we had not sufficiently addressed in our initial submission. Specifically, we feel the most important limitation of our conclusions is the inability to determine the relative contribution of B cell- versus cDC-derived IL-6 in modulating the antibody responses due to the alterations in cytokine production/consumption and alteration in relative cell abundance as part of our experimental approach. We have included this important caveat in the discussion of our findings.

Amendment:

- We have added text in the Discussion (lines 545-553) highlighting the limitation of our experimental approach to determine the relative importance of IFN-ɑ–cDC-derived versus B cell-derived IL-6 in modulating vaccine-induced antibody production.

Fig 6: The authors claim to measure spike-specific CD4 T cell responses. How do you know these are not simply responsive to the vector rather than spike protein?

Response:

Thank you for highlighting this important point. We agree that T cell AIM or ICS responses induced by ChAdOx1 nCoV-19 may be responsive to either the vector or the spike protein. We compared the CD4 T cell responses to ChAdOx1 nCoV-19 and ChAdOx1 GFP, resulting in negligible differences between the two ChAdOx1 stimulations (Fig S6B-C). A previous study (Wajja *et al.*, Lancet Infect Dis, 2024) reported that ChAdOx1-naive individuals also show detectable IFN-γ ELISpot responses towards ChAdOx1-GFP, highlighting the prevalence of cross-reactive ChAdOx1-responsive T cells even without prior ChAdOx1 exposure.

As such, our current experimental setup cannot distinguish between vector and transgene-specific CD4 T cell responses. We have thus amended our manuscript to refer to the AIM and ICS responses as ChAdOx1 nCoV-19-induced CD4 T cell responses instead of spike-specific responses.

Amendment:

- Added figures (Fig S6B-C) and revised text (lines 430-434, 458-459, 567) to more accurately refer to ChAdOx1 nCoV-19-induced CD4 T cell responses

Reviewer #2: (No Response)

Reviewer #3: Why do some donors don’t exhibit a response? The authors need to discuss this at least. Since the authors measured pre-existing S1-specific B cells, they should plot the antibody responses against those to demonstrate their point that “Our culture system appears to efficiently model a recall/boost response.”

Response:

Thank you for the suggestion. We have added Fig 1H to show the positive correlation between the pre-existing S1-specific B cells and antibody production.

The non-responder post-pandemic donors had some of the lowest frequencies of S1-specific B cells among the post-pandemic cohort. Given the correlation between the percentage of S1-specific B cells and antibody production and that the non-responding donors had lower S1-specific B cells, it is possible that non-responding donors have a sub-threshold memory B cell response. We have revised the manuscript to include these new data.

Amendment:

- Added Fig 1H and text (lines 284-285) on the correlation between percentage of S1-specific B cells at baseline and antibody production
- Added text (lines 286-287) on the lower frequency of S1-specific B cells in post-pandemic donors that did not produce specific antibody responses

Why did the vaccination suppressed unstimulated antibody production? The authors should at least discuss this in the text besides indicating that the samples will be excluded from the study.

Response:

Thank you for this suggestion. As requested by Reviewer 1, we have now measured total IgG levels in the system (Fig S2E). From this, we discovered that ChAdOx1 nCoV-19 stimulation suppressed non-specific (total) IgG production. This effect may have resulted from the increased metabolic requirements of activated B cells induced by ChAdOx1, resulting in resource competition among B cells.

In donors without spontaneous anti-S1 IgG production, this results in an increase in antigen-specific antibody production at the cost of non-specific antibody production. However, in donors with spontaneous anti-S1 IgG production, the spontaneous anti-S1 IgG production may be suppressed by the resource competition stimulated by ChAdOx1-induced B cell activation.

Amendment:

- Added Fig S4E and text (lines 255-257) on possible reason for why vaccination suppresses spontaneous antigen-specific antibody production

Why would pre-pandemic not produce a response?

This is an excellent question, and one for which we wish we had a comprehensive answer. The process of converting a naive B cell into a class-switched antibody-secreting cell requires multiple cell-cell interactions and signals – broadly speaking this would include a minimum of (1) initial cognate antigen capture by the BCR; (2) antigen processing and presentation on MHC II to cognate Tfh cells; (3) receipt of positive survival signals from Tfh cells; (4) receipt of signals to induce class-switch recombination; (5) receipt of signals to induce differentiation into a plasmablast. A prerequisite for step 1 to occur is the presence of naive B cells in the culture system that express a cognate BCR for the spike antigen. Based on our IgM production data (Fig 1C; lines 260-262) we believe this first requirement is only satisfied a portion of the time. Our intention in future studies is to investigate which of these steps are not being satisfied, thus resulting in a failure to induce responses from pre-pandemic donors. However, given the scope of the work required, we believe it falls beyond the current manuscript, which focuses on stimulation of memory responses.

Amendment:

- A brief sentence has been added to the discussion (lines 489-491) acknowledging that further work is required if this system is to be used for study of primary responses.

The IgM data is interesting but do the authors have some metadata about the post-pandemic cohort? Are they previously infected? Vaccinated? A better characterization of the cohort, if possible, is necessary in the methods section.

Response:

We agree that information on previous exposure would be helpful for the post-pandemic cohort. Unfortunately, records of previous vaccination/infection were not available for our cohort. We clarified this by adding text in the Methods section. However, the presence of S1-specific B cells likely indicates a form of prior exposure (either through infection or vaccination or both).

Amendment:

- Added text to clarify absence of information on previous exposure in post-pandemic cohort (lines 116-117)

Critically, since the authors posit that pre-pandemic cultures do not have enough time to class switch, have the authors incubated them longer or provided repeat immunization to see if they stimulate antibody production?

Response:

Thank you for this suggestion. We extended the culture time for pre-pandemic cultures up to 27 days of culture but still detected no anti-S1 IgG production in the pre-pandemic donors that we tested (Figure S2D). The experimental model needs dedicated optimisation to allow efficient induction of responses from naive donors, including to induce class switching. We hope that the Reviewer agrees with our conclusion that such optimisation reflects an entirely distinct project and is thus outside the scope of our current revised manuscript.

Amendment:

- Added Fig S2D and text (lines 246-248) on longer cultures of pre-pandemic donors
